# Multiple HPV genotype infection impact on invasive cervical cancer presentation and survival

**DOI:** 10.1371/journal.pone.0182854

**Published:** 2017-08-22

**Authors:** Maria Luiza Nogueira Dias Genta, Toni Ricardo Martins, Rossana V. Mendoza Lopez, José Carlos Sadalla, João Paulo Mancusi de Carvalho, Edmund Chada Baracat, José Eduardo Levi, Jesus Paula Carvalho

**Affiliations:** 1 Gynecological Oncology Department, Instituto do Câncer do Estado de São Paulo (ICESP), Faculdade de Medicina, Universidade de São Paulo, São Paulo, Brazil; 2 Virology Laboratory, Instituto de Medicina Tropical, Universidade de São Paulo, São Paulo, São Paulo, Brazil; 3 Centro de Investigação Translacional em Oncologia, Instituto do Câncer do Estado de São Paulo (ICESP), Faculdade de Medicina, Universidade de São Paulo, São Paulo, Brazil; 4 Division of Gynecologic Clinic, Hospital das Clínicas, Faculdade de Medicina, Universidade de São Paulo, São Paulo, Brazil; Universidade Estadual de Maringa, BRAZIL

## Abstract

**Background:**

Invasive cervical cancer (ICC) is the third most common malignant neoplasm affecting Brazilian women. Little is known about the impact of specific HPV genotypes in the prognosis of ICC. We hypothesized that HPV genotype would impact ICC clinical presentation and survival.

**Methods:**

Women diagnosed with ICC at the Instituto do Câncer do Estado de São Paulo (ICESP) between May 2008 and June 2012 were included in the study and were followed until December 2015. HPV genotype was detected from formalin-fixed paraffin-embedded (FFPE) tumor tissue samples using Onclarity™ system (BD Viper™ LT automated system).

**Results:**

292 patients aged 50±14 years were analyzed. HPVDNA was detected in 84% of patients. The HPV genotypes studied were: HPV16 (64%), HPV18 (10%), HPV33-58 (7%), HPV45 (5%), HPV31 (4%) and other high-risk HPV genotypes (11%). HPV genotypes showed different distributions regarding histological type and clinical stage. Patients were followed for 35±21 months. The overall survival at 5 years after diagnosis of cervical cancer was 54%. Age, clinical staging, histological type and multiple HPV genotypes infection detected in the same tumor specimen were associated with poorer overall survival on multivariate Cox proportional hazard analysis (p<0.05). No specific HPV genotype affected survival.

**Conclusion:**

Multiple HPV genotype infection was associated with poorer ICC survival in our study, compared with single genotype infection. HPV genotyping from FFPE tumor tissue using an automated assay such as the Onclarity BD™ assay provides a simpler alternative for routine clinical use.

**Impact:**

This is the largest study employing an automated HPV genotyping assay using FFPE of ICC. Multiple HPV genotype infection adversely influenced survival.

## Introduction

Despite several years have passed since the implementation of cervical cancer screening, invasive cervical cancer (ICC) continues to be a global health burden. Human Papillomavirus (HPV) has been identified as the etiologic cause of ICC[1]. The natural history of HPV infection that leads to the development of ICC offers an opportunity for ICC prevention, which can be accomplished through HPV screening from cervicovaginal specimens. ICC prevention can also be provided through HPV vaccination. Nevertheless, ICC remains prevalent, largely due to incomplete screening and vaccination coverage[2]. In Brazil, 16,340 new cases are estimated in 2016[3]. Survival rate among developing countries (50% in 5 years) is lower than in developed countries (59–69%), partially because diagnosis is made at later stages[4].

HPV genotypes differ in their oncogenic potential, thus being classified as high and low risk genotypes[5]. Studies that addressed the impact of HPV genotypes on survival are heterogeneous, specifically with respect to genotyping method (serological vs molecular), population studied and whether ICC was confirmed as the primary cause of death[6–12]. In addition, as we expect that the incidence of HPV 16/18-associated ICC will reduce in countries that adopt vaccination, it is important to understand what is the role of the HPV genotypes not covered by the new nonavalent HPV9 vaccine, on disease presentation and survival. While multiple HPV genotypes have been described in ICC specimens, the oncogenic implication of co-infection remains controversial[13, 14].

In the present study, consecutive ICC patients referred to the Instituto do Câncer do Estado de São Paulo (ICESP) between 2008 and 2012 were followed until December 2015. HPV genotype was determined from formalin-fixed paraffin-embedded (FFPE) tumor tissue samples. We hypothesized that HPV genotype would impact ICC clinical presentation and survival.

## Methods

### Study population

Women referred to the Instituto do Câncer do Estado de São Paulo (ICESP) with a diagnosis of ICC from March 2008 to June 2012 that had FFPE blocks available were included. All patients were staged according to the International Federation of Gynecology and Obstetrics (FIGO) staging system[15]. Patients were followed until December 2015. The study protocol was approved by the Comitê de Ética e Pesquisa da Faculdade de Medicina da Universidade de São Paulo (#34718). Informed consent was waived by the ethics committee. Data was acessed anonymously.

### HPV detection and typing

Three 10 micron-thick tissue sections were taken from each FFPE block. The first and last sections were stained with hematoxylin and eosin (H&E) to confirm the diagnosis of ICC. Standard measures to avoid cross-contamination were taken during tissue sectioning and processing. HPV type was determined using BD Onclarity™ HPV Assay (BD Diagnostics, Sparks, USA). BD Onclarity™ is a real-time PCR assay that detects HPV type-specific E6 and E7 genes. It simultaneously detects 14 high-risk HPV types, and can provide genotyping information on six individual types (HPV 16, 18, 31, 45, 51 and 52), reporting the remaining HPV types in three distinct groups (33 and 58; 56, 59 and 66; and 35, 39 and 68). Each FFPE tissue sample was extracted using the automated workflow on the Viper™ LT system. The tissue section was combined with 0.5 mL of distilled water and added directly to a tube with pierce able cap containing a proprietary diluent. The sample was then lysed directly using the Viper™ LT Pre-warm station before being transferred onto the deck of the instrument where it underwent automated sample processing and PCR detection. Briefly, the DNA was extracted using BD FOX™ magnetic particles and the eluate-containing DNA was used to set up three PCR genotyping reactions: G1 detects HPV 16, HPV 18 and HPV 45 plus the internal beta globin control; G2 detects HPV 31, HPV 33–58 and HPV 56-59-66 plus the internal beta globin control; G3 detects HPV 51, HPV 52 and HPV 35_39_68 plus the internal beta globin control. After 40 PCR cycles, any Ct score for a specific type and/or the internal beta globin control was considered positive for that channel[16].

### Statistical analysis

Statistical analyses were performed using SPSS for Windows v.18. Continuous variables are described as mean ± standard deviation and categorical variables as absolute and relative frequencies. Chi-square test was used to compare the distribution of HPV genotype among clinical and pathological parameters. Kaplan-Meier method was used for survival analysis. Survival curves were compared using the log-rank test. Univariate Cox regression analysis was used to test HPV genotype, age, clinical staging and histological type. Variables showing a P value<0.2 in the univariate analysis were included in a multiple Cox regression model. Interaction between HPV genotype and FIGO and histological type were also tested in the multiple Cox regression model. For the remainder analysis, A p-value of <0.05 was considered statistically significant.

## Results

Viable human DNA was successfully detected from 292 formalin-fixed paraffin-embedded tumor samples. Human DNA viability was confirmed through the detection of human beta-globin DNA. Viable human DNA was not detected in 27 subjects who were excluded from subsequent analysis. Included patients were aged 50±14 years (range = 17–87 years) (Table 1).

**Table 1:** Clinical and pathological parameters of all subjects with viable human DNA.

	*n*	*(%)*	*5-year survival (%)*
*Age*, *years*			
***≤30***	*31*	*10*.*6*	*43*.*2*
***31–40***	*40*	*13*.*7*	*74*.*7*
***41–50***	*81*	*27*.*7*	*64*.*8*
***51–60***	*71*	*24*.*3*	*40*.*5*
***61–70***	*43*	*14*.*7*	*59*.*0*
***>70***	*26*	*8*.*9*	*44*.*0*
*FIGO stage*			
***I-IB1***	*98*	*33*.*6*	*78*.*9*
***IB2-IIB***	*143*	*49*.*0*	*52*.*8*
***IIIA-IVB***	*51*	*17*.*5*	*23*.*4*
*Histological type*			
***Squamous cell carcinoma (SCC)***	*217*	*74*.*3*	*53*.*9*
***Adenocarcinoma (ADC)***	*60*	*20*.*5*	*72*.*2*
***Other***	*15*	*5*.*1*	*19*.*4*
*Tumor Size*			
***<4 cm***	*104*	*35*.*6*	*77*.*5*
***> = 4cm***	*135*	*46*.*2*	*50*.*2*
***Not reported***	*53*	*18*.*2*	*31*.*8*
*Primary treatment*			
***Surgery***	*50*	*17*.*1*	*88*.*6*
***Surgery + Adjuvant Radiotherapy***	*74*	*25*.*3*	*74*.*9*
***Radiotherapy +/- Chemotherapy***	*152*	*52*.*1*	*38*.*5*
***Paliative***	*16*	*5*.*5*	*28*.*6*
*Recurrence*			
***Yes***	*120*	*41*.*1*	*19*.*2*
***No***	*171*	*58*.*6*	*88*.*2*
*Smoking*			
***Yes***	*130*	*44*.*5*	*53*.*0*
***No***	*149*	*51*.*0*	*59*.*4*
***Not available***	*13*	*4*.*5*	*47*.*7*

HPV DNA was detected from 246 of the 292 samples with viable human DNA (84.2%). The most frequent HPV types were HPV16 (64%), HPV18 (10%), HPV33-58 (7%), HPV45 (5%), HPV31 (4%) and other types of HPV(HPV51, HPV52, HPV56-59-66 and HPV35-39-68) (11%). The presence of more than one HPV genotype in tumor sample occurred in 11 cases (4%). Among all 11 multiple-genotype infected subjects, HPV16 was present in 10. (S1 Table)

The distribution of clinical and histopathological parameters according to the most frequent HPV genotypes is shown in Table 2. Adenocarcinoma was associated with a higher proportion of HPV18 cases (37.5% of all HPV18+samples) than of HPV16 cases (19.1% of all HPV16+ samples) (p = 0.032). HPV33-58 and HPV31 were detected only among subjects with squamous cell carcinoma.

**Table 2:** Distribution of the most frequent HPV genotypes according to clinical and pathological variables.

*Parameter*	*HPV 16*	*HPV 18*	*HPV31*	*HPV33-58*	*HPV45*	*HPVother*	*Total*	*p*
***Age*** ***Mean (DP)***	*49(14*.*1)*	*50(11*.*7)*	*55(13*.*6)*	*53(12*.*4)*	*45(13*.*4)*	*54(14*.*9)*	*50(13*.*9)*	*0*.*651*
***≤30 ys*, *(%)***	*21 (13*.*3)*	*1 (4*.*2)*	*0 (0)*	*1 (5*.*9)*	*1 (8*.*3)*	*1 (3*.*9)*	*25 (10*.*1)*	
***31–40 ys*, *(%)***	*24 (15*.*3)*	*3 (12*.*5)*	*1 (10*.*0)*	*1 (5*.*9)*	*3 (25*.*0)*	*3 (11*.*5)*	*35 (14*.*2)*	
***41–50 ys*, *(%)***	*41 (26*.*1)*	*8 (33*.*3)*	*3 (30*.*0)*	*6 (35*.*3)*	*6 (50*.*0)*	*7 (26*.*9)*	*71 (28*.*8)*	
***51–60 ys*, *(%)***	*34 (21*.*6)*	*8 (33*.*3)*	*3 (30*.*0)*	*6 (35*.*3)*	*1 (8*.*3)*	*7 (26*.*9)*	*59 (24*.*0)*	
***61–70 ys*, *(%)***	*27 (17*.*2)*	*3 (12*.*5)*	*1 (10*.*0)*	*1 (5*.*9)*	*0 (0)*	*4 (15*.*4)*	*36 (14*.*6)*	
***>70 ys*, *(%)***	*10 (6*.*4)*	*1 (4*.*2)*	*2 (20*.*0)*	*2 (11*.*8)*	*1 (8*.*3)*	*4 (15*.*4)*	*20 (8*.*1)*	
***Histology***								***0*.*044***
***SCC*, *n(%)***	*118 (75*.*1)*	*13 (54*.*1)*	*10 (100*.*0)*	*17 (100*.*0)*	*9 (75*.*0)*	*23 (88*.*5)*	*190 (77*.*2)*	
***ADC*, *n(%)***	*30 (19*.*1)*	*9 (37*.*5)*	*0(0)*	*0(0)*	*3 (25*.*0)*	*2 (7*.*7)*	*44 (17*.*8)*	
***Other*, *n(%)***	*9 (5*.*7)*	*2 (8*.*3)*	*0(0)*	*0(0)*	*0(0)*	*1 (3*.*9)*	*12 (4*.*9)*	
***FIGO***								***0*.*025***
***I-IB1*, *n(%)***	*54 (34*.*4)*	*7 (29*.*2)*	*5 (50*.*0)*	*4 (23*.*5)*	*2 (16*.*7)*	*9 (34*.*6)*	*81 (32*.*9)*	
***IB2-IIB*, *n(%)***	*88 (56*.*0)*	*11 (45*.*8)*	*4 (40*.*0)*	*6 (35*.*3)*	*9 (75*.*0)*	*11 (42*.*3)*	*129 (52*.*4)*	
***IIIA-IVB*, *n(%)***	*15 (9*.*5)*	*6 (25*.*0)*	*1 (10*.*0)*	*7 (41*.*2)*	*1 (8*.*3)*	*6 (23*.*1)*	*36 (14*.*6)*	
***Total(n)***	*157 (100)*	*24 (100)*	*10 (100)*	*17 (100)*	*12 (100)*	*26 (100)*	***246 (100)***	

HPVother = HPV51, HPV52, HPV56-59-6 and HPV35-39-68

Patients were followed for 35±21months (range = 1–86 months). The cumulative overall survival at 5 years after diagnosis of cervical cancer was 54%. There were 88 deaths during the study period and 103 recurrences (42%), subdivided into three groups: local (13%), regional (31%) and distant (56%). (Table 1)

**Fig 1:**
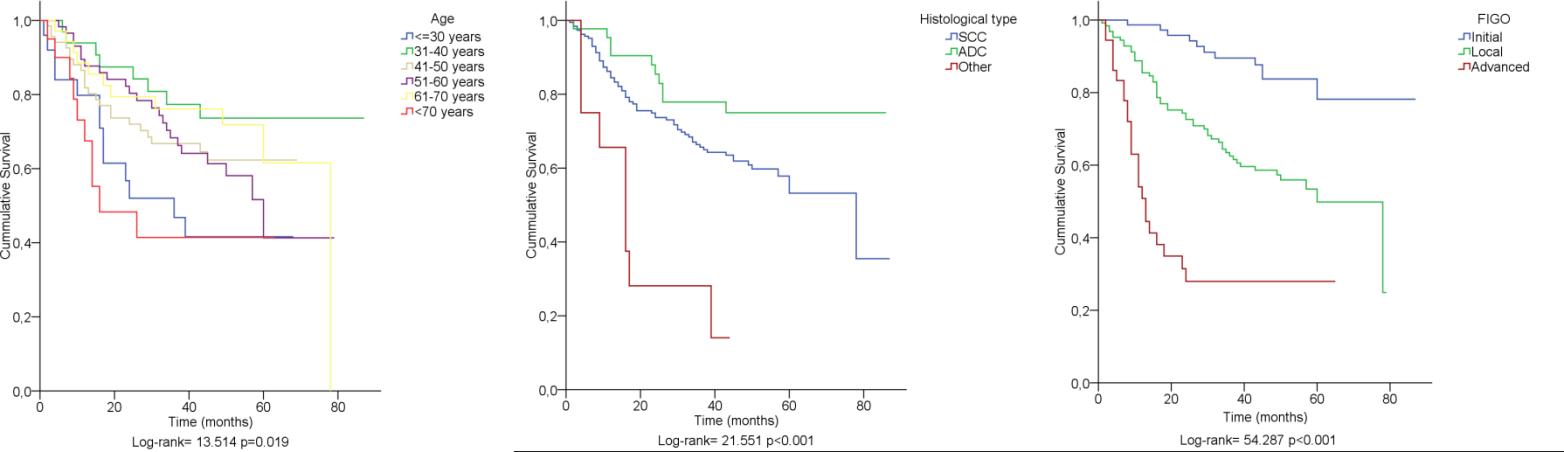
Kaplan-Meier survival curves stratified by age (left panel), clinical stage (middle panel) and histological type (right panel).

Patients aged ≤ 30 and >70 years had poorer survival at univariate analysis (p<0.01). Patients diagnosed at Initial stages had better survival when compared with patients at more advanced stages (p<0.001) (Table 3, Fig 1). At multivariate analysis, patients over 70 years had poorer prognosis (p<0.01) (Table 4).

**Table 3:** Five-year survival rate according to clinical, histopathological and HPVgenotype variables.

*Prognostic parameters*	*Number of deaths/Total*	*5-year survival (%)*	*Hazard Ratio*	*95% CI*	*p-value*
***Age*, *years***					*0*.019*
***≤30***	*13/25*	*41*.*6*	*3*.*296*	*1*.*352–8*.*036*	*0*.*009*
***31–40***	*8/35*	*73*.*7*	*1*.*0*	*-*	*-*
***41–50***	*23/71*	*62*.*3*	*1*.*793*	*0*.*794–4*.*048*	*0*.*160*
***51–60***	*23/59*	*41*.*3*	*1*.*896*	*0*.*843–4*.*268*	*0*.*122*
***61–70***	*11/36*	*61*.*6*	*1*.*424*	*0*.*571–3*.*554*	*0*.*448*
***>70***	*10/20*	*41*.*4*	*3*.*815*	*1*.*490–9*.*770*	*0*.*005*
***FIGO stage***					*<0*.001*
***I-IB1***	*11/81*	*78*.*2*	*1*.*0*	*-*	*-*
***IB2-IIB***	*53/129*	*49*.*8*	*3*.*536*	*1*.*845–6*.*776*	*<0*.*001*
***IIIA-IVB***	*24/36*	*28*.*0*	*10*.*452*	*5*.*072–21*.*538*	*<0*.*001*
***Histological type***					*<0*.001*
***Squamous cell carcinoma***	*69/190*	*53*.*2*	*1*.*923*	*0*.*989–3*.*735*	*0*.*054*
***Adenocarcinoma***	*10/44*	*75*.*0*	*1*.*0*	*-*	*-*
***Other***	*9/12*	*14*.*1*	*6*.*937*	*2*.*789–17*.*250*	*<0*.*001*
***HPV DNA Positive***					*0*.132*
***HPVsingle***	*66/199*	*59*.*3*	*1*.*0*	*-*	*-*
***HPVmultiple***	*6/11*	*35*.*0*	*1*.*877*	*0*.*813–4*.*335*	*0*.*140*
***HPV genotype***					*0*.225*
***HPV 16/18 +***	*62/181*	*57*.*3*	*1*.*0*	*-*	*-*
***HPV 16/18 -***	*26/65*	*49*.*7*	*1*.*326*	*0*.*837–2*.*101*	*0*.*229*

*p from Log-rank Test. 95%CI = 95% confidence interval

**Table 4:** Multivariate predictors of overall survival according to Cox proportional hazard model.

*Prognostic parameters*	*5-year survival (%)*	*Hazard Ratio*	*95% CI*	*p-value*
***Age*, *years***				
***≤30***	*41*.*6*	*2*.*542*	*0*.*909-7-110*	*0*.*075*
***31–40***	*73*.*7*	*1*	*-*	*-*
***41–50***	*62*.*3*	*1*.*922*	*0*.*745–4*.*957*	*0*.*177*
***51–60***	*41*.*3*	*2*.*038*	*0*.*794–5*.*230*	*0*.*139*
***61–70***	*61*.*6*	*1*.*517*	*0*.*542–4*.*245*	*0*.*427*
***>70***	*41*.*4*	*3*.*196*	*1*.*069–9*.*553*	*0*.*038*
***FIGO stage***				
***I-IB1***	*78*.*2*	*1*.*0*	*-*	*-*
***IB2-IIB***	*49*.*8*	*4*.*061*	*1*.*797–9*.*177*	*0*.*001*
***IIIA-IVB***	*28*.*0*	*11*.*041*	*4*.*308–28*.*300*	*<0*.*001*
***Histological type***				
***Squamous cell carcinoma***	*53*.*2*	*1*.*213*	*0*.*581–2*.*533*	*0*.*606*
***Adenocarcinoma***	*75*.*0*	*1*.*0*	*-*	*-*
***Other***	*14*.*1*	*3*.*501*	*1*.*294–9*.*470*	*0*.*014*
***HPV infection***				
***HPVsingle***	*59*.*3*	*1*.*0*	*-*	*-*
***HPVmultiple***	*35*.*0*	*2*.*522*	*1*.*050–6*.*059*	*0*.*039*

95%CI = 95% confidence interval

**Fig 2:**
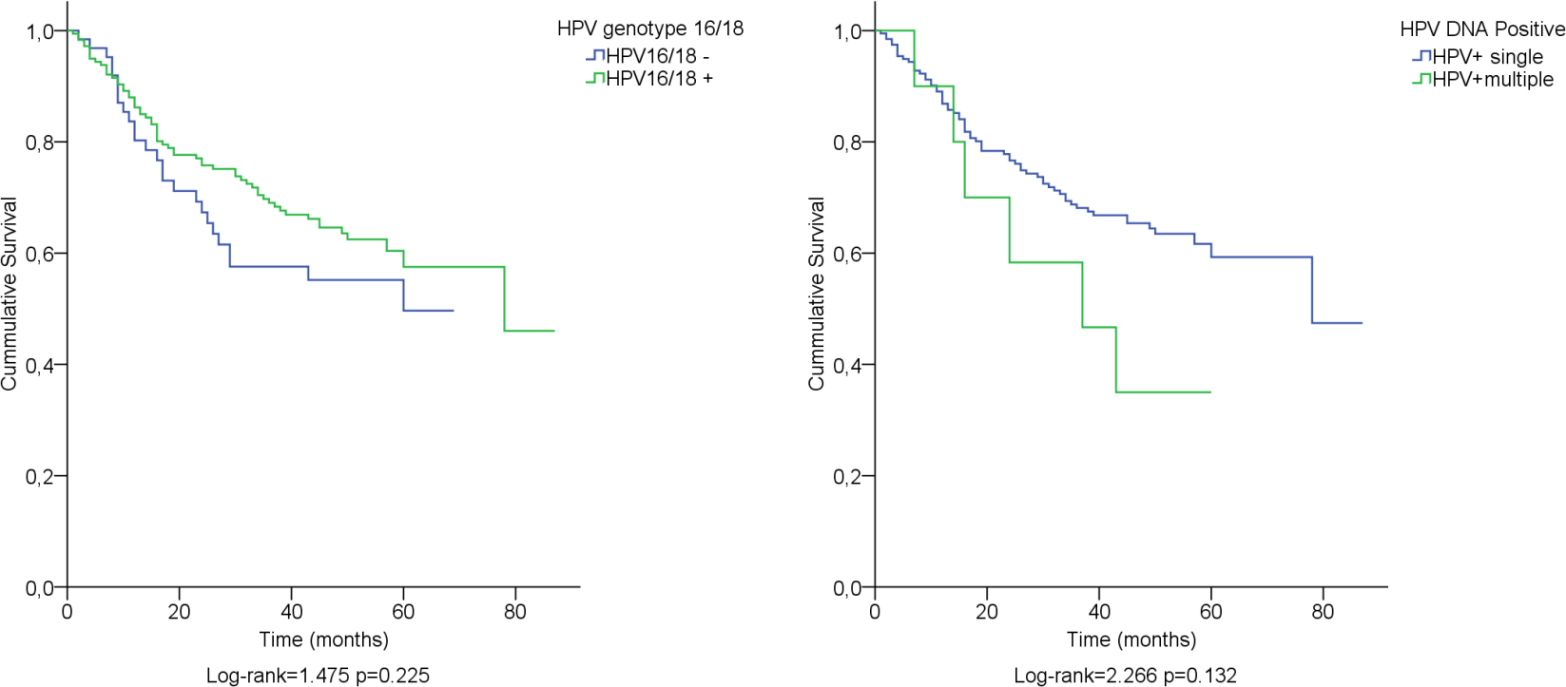
Kaplan-Meier survival curves stratified by the presence of HPV genotype 16/18 (left panel). ICC survival stratified by single or multiple HPV infection (right panel).

The most common HPV subtypes (HPV16 and 18) did not influence prognosis compared to the remaining HPV subtypes (p = 0.225) (Fig 2). Multiple HPV infection detected in the same sample was associated with poorer overall survival (p = 0.039) (Table 4 and Fig 2).

Age at diagnosis, clinical stage, histological type and HPVsingle or HPVmultiple were analyzed using a Cox regression multivariate model. No individual HPV genotypes were differentially associated to survival (Table 4).

## Discussion

In the present study, 292 patients with ICC had FFPE tumor samples submitted for HPV DNA genotyping (Onclarity BD™ HPV assay). The patients were followed at the Instituto do Câncer do Estado de São Paulo for a mean of 35 months. The most important findings were: 1. Onclarity BD™ HPV assay was able to detect HPVDNA in 84% of FFPE tumor samples and showed similar contribution of the eight most commonly reported HPV genotypes (HPV16, 18, 31, 33, 35, 45, 52 and 58) as verified worldwide[17]; 2. HPV genotypes displayed different distribution according to histological type and clinical stage at diagnosis; 3. age, clinical staging, histological type and multiple HPV infection were independent predictors of survival.

The present study is the first that employed the Onclarity BD ™ assay for HPV genotyping from FFPE ICC tumor samples. Previous studies used the Onclarity BD™ assay in cervical intraepitelial neoplasia grades 2 or 3 FFPE samples and found HPV DNA in 90%[18]. In line with these previous reports, the present study was able to detect HPV DNA in 84% of tumoral samples. De Sanjose et al was able to detect HPV DNA in 85% of 10,575 FFPE tumor samples using non-automated genotyping methods[17]. Formalin fixation may cause extensive DNA damage, including cross-linking and fragmentation, and may decrease HPV DNA detection accuracy. Successful amplification of HPV sequences from archival FFPE specimens has been shown to be inversely correlated to the length of the PCR amplicon method. In addition, specimen age may contribute to nucleic acid degradation[16]. The high detection rate of HPV DNA observed in the present study demonstrates that BD Onclarity™ assay is an attractive automated alternative for HPV genotyping from FFPE tumor samples.

The prevalence of the eight most common HPV genotypes in the present study (HPV16, 18, 31, 33, 35, 45, 52 and 58) agrees with previous studies[19, 20]. HPV 16 and 18 were observed in 74% of cases and would be covered by the tetravalent (quadrivalent) vaccine currently used in Brazil. Based in the observed HPV genotypes found in our population, the new nonavalent vaccine will be able to prevent up to 90% of ICC. However, the real impact of the nonavalent vaccine will depend on vaccination coverage and vaccine efficacy[21].

The average age at ICC diagnosis (50±14ys) observed in the present study was similar to those reported in North America (51±16ys), Asia (51±13ys) and Oceania (49±14ys), but lower than in Europe (54 ±14ys)[17]. The regional average age difference may reflect, in part, the effectiveness of cervical cancer screening programs. In countries with higher income and more organized screening programs, the average age of ICC diagnosis tends to be higher and at earlier clinical stages[22]. It may also reflect the distribution and the oncogenic potential of each individual HPV genotype within a given population. It has been shown that the average age at ICC diagnosis is lower for HPV16, 18 and 45 than other high-risk types[17].

Approximately 11% of the patients evaluated in this study were aged ≤ 30 years, which was associated to poorer prognosis at univariate Cox-proportional hazards (p<0.01). Cervical cancer screening in Brazil is based on conventional cervicovaginal cytology and is recommended for women of 25–64 years[3]. Screening coverage in Brazil remains suboptimal[23]. In addition, the relatively late age of initial cervical cancer screening may be inadequate to diagnose preneoplastic lesions and ICC among young women. The elevated proportion of ICC among young women in our study challenges the relatively late cervical cancer screening onset and raises concern about screening coverage.

In the present study, individual HPV genotypes were not associated with prognosis, which is in line with previous observations[8, 24, 25]. On the other hand, multiple HPV infection was an independent predictor of poorer survival in the present study. The reasons why multiple HPV infection affects survival are not fully understood. The presence of multiple HPV genotypes may increase the length of persistent HPV infection and possibly the risk of carcinogenesis[26]. Few studies have described the association of multiple HPV infections and cervical cancer, and the results were inconsistent[27]. Some studies have suggested a possible role for multiple HPV infection in the development or progression to neoplasia[14]. In contrast, other studies have shown that the development of cervical pre-invasive lesions or invasive cancer in women with multiple HPV infection genotypes of HPV was similar to those infected by a single HPV genotype[1, 28]. Our results suggest that the detection of multiple HPV genotyping among ICC patients may improve survival prediction.

Our study has some limitations. Firstly, HPV DNA negative samples (16% of our sample) may represent false negative results which may have masked associations between HPV genotype and prognosis. However, HPV DNA detection rate in our study (84%) was comparable to previous studies[16, 17] The use of additional detection methods was not feasible to address this issue in our study but could have improved HPV DNA detection. Secondly, only 11 subjects presented multiple HPV genotype infection. The HPV DNA genotype detection method used groups some genotypes together. When a patient was positive for a HPV genotype group, it was not possible to exclude infection by more than one HPV genotype within that group. Further studies that address the role of multiple HPV infection on the development and prognosis of ICC are warranted. Thirdly, the number of patients with multiple HPV infection was relatively small. Lastly, the study was not adequately powered to test the influence of the different HPV genotypes on clinical stage, age at diagnosis and recurrence. Larger studies are needed to address these issues.

## Conclusion

Multiple HPV genotype infection was associated with poorer ICC survival in our study, compared with single genotype infection. HPV genotyping from FFPE tumor tissue using an automated assay such as the Onclarity BD™ assay provides a simpler alternative for routine clinical use.

## Supporting information

S1 Table HPV DNA genotype frequency in the population studied. (DOCX)
