# Supplementary material for: Multiple HPV genotype infection impact on invasive cervical cancer presentation and survival
Source: PLoS One. 2017 Aug 22;12(8):e0182854. doi: 10.1371/journal.pone.0182854 (PMC5567480; doi:10.1371/journal.pone.0182854)
Supplement: S1 Table — (DOCX) [file pone.0182854.s001.docx]

Supporting information:

S1 Table: HPV DNA genotype frequency in the population studied

| HPV DNA | | | Frequency (n) | | Percent (%) | | Cumulative Percent (%) | |
| --- | --- | --- | --- | --- | --- | --- | --- | --- |
|  | HPV16 | 149 | | 51,0 | | 51,0 | |  |
|  | HPV16,18 | 1 | | ,3 | | 51,4 | |  |
|  | HPV16,18,59-56-66 | 1 | | ,3 | | 51,7 | |  |
|  | HPV16,31 | 3 | | 1,0 | | 52,7 | |  |
|  | HPV16,31,59-56-66 | 1 | | ,3 | | 53,1 | |  |
|  | HPV16,33-58,45 | 1 | | ,3 | | 53,4 | |  |
|  | HPV16,33,58 | 1 | | ,3 | | 53,8 | |  |
|  | HPV16,39-68-35 | 1 | | ,3 | | 54,1 | |  |
|  | HPV16,59-56-66 | 1 | | ,3 | | 54,5 | |  |
|  | HPV18 | 25 | | 8,6 | | 63,0 | |  |
|  | HPV18,31 | 1 | | ,3 | | 63,4 | |  |
|  | HPV31 | 11 | | 3,8 | | 67,1 | |  |
|  | HPV33-58 | 17 | | 5,8 | | 72,9 | |  |
|  | HPV39-68-35 | 9 | | 3,1 | | 76,0 | |  |
|  | HPV45 | 14 | | 4,8 | | 80,8 | |  |
|  | HPV51,59-56-66 | 1 | | ,3 | | 81,2 | |  |
|  | HPV52 | 6 | | 2,1 | | 83,2 | |  |
|  | HPV59-56-66 | 10 | | 3,4 | | 86,6 | |  |
|  | HPVnegativo | 39 | | 13,4 | | 100,0 | |  |
|  | Total | 292 | | 100,0 | |  | |  |
